# Eco-design and life cycle characterization of fiber-metal laminates for sustainable manufacturing and resource efficiency

**DOI:** 10.1038/s41598-026-50250-6

**Published:** 2026-05-07

**Authors:** Anand Pai, Marcos Rodriguez-Millan, Satish Shenoy Baloor, Suhas Yeshwant Nayak, J. P. Jaideep

**Affiliations:** 1 Departamento de Ingeniería Mecánica, Universidad Carlos III de Madrid, Avenida de la Universidad, 30 (Edificio Sabatini), 28911 Leganés, Madrid Spain; 2 Manipal Institute of Technology, Manipal Academy of Higher Education, Manipal, India

**Keywords:** Fiber-metal laminates, Life cycle assessment, Woven fabrics, Aluminium alloys, Cradle-to-gate, Sustainable manufacturing, Climate action, Engineering, Environmental sciences, Materials science

## Abstract

Fiber-metal laminates (FMLs) are high-performance hybrid materials that integrate metal layers with fiber-reinforced polymer (FRP) composites to achieve superior strength-to-weight ratios. This study presents a comparative cradle-to-gate Life Cycle Assessment (LCA) of six distinct FML configurations, focusing on the environmental influence of fiber selection and metal surface treatment. The FMLs utilized AA2024-T3 aluminium alloy as facing layers, integrated with various high-performance fabrics: Basalt, Aramid, Zylon, and Innegra. A significant contribution of this research is the development of original inventory datasets for these specialized fiber fabrics, which were previously limited in existing databases. Using OpenLCA software with the ecoinvent 3.12 database and the ReCiPe 2016 Midpoint (H) method, the study integrates detailed surface treatment processes—specifically chemical anodizing and mechanical abrasion—directly into the LCA framework. The analysis identifies critical environmental hotspots, demonstrating that the choice of interfacial treatment and fiber type significantly dictates the sustainability profile of the laminate. The findings underscore the necessity of eco-design strategies in FML development, advocating for the substitution of high-impact components, the adoption of low-impact surface treatments, and the integration of energy-efficient manufacturing to advance circular economy pathways for hybrid composite systems.

## Introduction

Fiber-Metal Laminates represent an advanced class of hybrid structural materials that integrate the beneficial characteristics of metals and fiber-reinforced composites into a unified architecture^1,2^. Constructed from alternating layers of thin metallic sheets-most commonly aluminium alloys-and FRPs, FMLs achieve a synergistic balance of properties unattainable by either constituent material alone^3–6^. The metallic layers contribute to impact resistance, bearing strength, and corrosion protection, while the composite layers provide high specific stiffness, fatigue resistance, and crack-arresting capability^4,7–9^. This unique architecture imparts FMLs with a combination of lightweight performance, superior damage tolerance, extended fatigue life, and resistance to environmental degradation, making them highly attractive for structurally demanding sectors such as aerospace, automotive, marine, and defense engineering^6,10–12^. Applications range from aircraft fuselage skins and wing panels to automotive crash structures and marine hull components, where structural efficiency, safety, and durability are of paramount importance^13,14^. Hybrid materials, particularly FMLs, offer a superior balance of high specific strength, fracture toughness, and fatigue resistance compared to monolithic alloys. To further optimize these properties, recent research has focused on advanced manufacturing interventions. These include enhancing interfacial adhesion through specialized surface treatments, augmenting the polymer matrix via nanofiller resin modification, and implementing functional ply treatments prior to layup to ensure structural integrity under extreme loading conditions^15^.

### Anodizing treatment for enhanced interfacial adhesion

In recent years, substantial research has focused on improving the interfacial adhesion between metallic plies—particularly aluminium alloys—and fibre-reinforced composite layers. Surface modification strategies used for this purpose can be broadly classified into four categories: (i) mechanical methods such as surface abrasion; (ii) chemical treatments including nitric acid etching, alkaline degreasing or ferric sulphate/acid etching; (iii) electrochemical approaches such as anodizing; and (iv) advanced texturing techniques like electric-discharge-machining (EDM)-based surface patterning^15–17^. These methods collectively aim to enhance surface roughness, increase surface energy, and improve chemical compatibility, thereby strengthening the fibre–metal interface. Fiore et al.^18^ explored the impact of tartaric sulfuric acid (TSA) anodizing on AA5083 aluminium alloy to improve both adhesion strength and corrosion resistance in composite joints designed for nautical applications. The TSA anodizing process generated nanostructured anodic layers that significantly enhanced joint performance, especially in chloride-rich environments. A subsequent alkaline post-treatment (using sodium hydroxide) further increased resin uptake and mechanical interlocking, resulting in up to an 83% improvement in shear strength with basalt fibre reinforcement. Moreover, silane coupling agent treatment of glass and basalt fibres effectively improved fibre-matrix adhesion, contributing to the overall mechanical robustness of the joints. Balkundhi et al.^19^ reported that surface treatments significantly influence carbon fiber-reinforced aluminium laminates. Sulfuric acid anodizing produced nanostructured hydrophilic surfaces with the highest surface energy, improving tensile and flexural strengths by 61% and 104%, respectively. In contrast, mechanical abrasion and electric discharge texturing increased roughness but yielded hydrophobic, lower-energy surfaces. The study highlights surface engineering as key to enhancing laminate strength and delamination resistance.

### Environmental sustainability of sandwich structures

As the adoption of sandwich structures including the FMLs accelerates, the focus must extend beyond their mechanical superiority to encompass their environmental sustainability. The hybrid and multi-material nature of these laminates poses challenges for sustainability assessments, particularly in relation to raw material extraction, energy-intensive manufacturing, recyclability, and end-of-life management. Unlike monolithic metals or composites, disassembly and recycling of FMLs are technologically complex, with potential trade-offs between in-service benefits (e.g., reduced fuel consumption through weight savings) and production-phase burdens (e.g., greenhouse gas emissions, resource usage). To capture these trade-offs comprehensively, it becomes essential to evaluate FMLs across their entire life cycle-from material sourcing and processing, through laminate fabrication and service life, to maintenance, disposal, reuse, or recycling routes. In this context, Life Cycle Assessment (LCA) emerges as a critical methodology^20^. By providing a systematic and quantitative framework, LCA enables researchers and engineers to measure, compare, and optimize the environmental footprint of FMLs while ensuring that their mechanical and operational advantages are not achieved at the expense of long-term ecological sustainability. Over the past two decades, the LCA of composite structures in engineering and civil applications have been widely studied for different polymer matrices like bio-derived thermoset resins^21^ ; bio-based fibers like flax, ramie^22^, wheat straw^23^, olive fiber^24^; recycled carbon fibers^22,25^; thermoplastic like polypropylene and low-density polyethylene^26^, poly-lactic acid^27^, super absorbent polymers for cementitious composites^28^. Krzak et al.^29^ evaluated the environmental impact of epoxy resin systems for cryogenic applications, comparing the base resin with a bromine-based variant. The base epoxy resin was bisphenol-A cured using 4,4′-Methylenedianiline (MDA) hardener in a mix ratio of 100:27 by weight. The study revealed that the bromine-based resin caused greater environmental impact, through steeper global warming potential, human toxicity, water consumption, and marine pollution, largely due to its energy-intensive production in terms of heat and electricity. The bisphenol-A variant showed a lower overall carbon footprint, with marine transport as the primary emission source. Similar works^30,31^ on LCA of epoxy based resins indicate the requirements to prioritize low-impact resin systems in sustainable development of composites.

There have been some recent advancements in the environment sustainability analysis of FMLs. Braga et al.^32^ utilized a cradle-to-gate methodology to evaluate twelve configurations of fiber-metal laminates featuring aluminium skins and various polymer matrices reinforced with sisal and coir fibers. The analysis, conducted through the Open Life Cycle Assessment software and the ecoinvent database, revealed that bio-based polyurethane offered the lowest environmental burden. Conversely, the study identified that the mechanical and chemical preparation of the aluminium skins and the specific fiber treatments were the primary contributors to the overall ecological impact.^33^ investigated aluminium–core hybrid laminates incorporating core materials derived from waste coir fibers. The authors found that usage of castor-oil-based polyurethane enabled strong interfacial bonding through mechanical abrasion of the aluminium surface alone, eliminating the need for chemical surface treatments required by synthetic resins. This reduction in processing steps, combined with the use of natural waste fibers, enhanced the eco-efficiency of the laminate without compromising mechanical performance. The application of life cycle assessment to fiber-metal laminates provides a comprehensive assessment of their environmental profile, encompassing raw material sourcing, laminate curing, in-service performance, and end-of-life treatment. It enables comparisons among different laminate subtypes and with conventional materials such as aluminium, titanium, and composites. Life cycle assessment also influences design choices related to fiber volume fraction, ply orientation, and metal thickness, which are critical for environmental optimization. Specifically, by increasing the fiber-reinforced polymer content and reducing the metallic layer thickness, a significant reduction in total structural mass is achieved. In aerospace applications, this mass reduction directly translates to lower thrust requirements and reduced fuel consumption during the operational phase, as less energy is required to overcome inertia and maintain lift. Furthermore, this methodology can identify trade-offs between durability, recyclability, and operational fuel savings that are particularly relevant in aerospace, automotive, and marine sectors. Beyond comparative evaluation, LCA functions as a framework for aligning FML development with regulatory requirements and sustainability objectives, while also supporting the exploration of greener alternatives such as bio-based resins, recyclable thermoplastics, and hybrid architectures designed for simplified disassembly.

Despite these findings, a comprehensive environmental comparison involving high-performance synthetic fibers and the specific energy overhead of the associated manufacturing techniques remains sparse. While previous studies have established that LCA can influence design choices—such as fiber volume fraction and ply orientation to reduce structural mass—most literature focuses on either raw material sourcing or simplified, theoretical fabrication models. Furthermore, existing studies predominantly cover natural fibers which, while sustainable, often lack the mechanical threshold required for high-velocity impact applications. Consequently, there is a critical need for a framework that identifies the trade-offs between durability, recyclability, and the granular impacts of post-processing for structural-grade materials, even at the laboratory scale.

The present study addresses this gap by extending the cradle-to-gate LCA framework to novel configurations of FML stackups fabricated under controlled laboratory conditions. Unlike previous works that focus primarily on natural fibers or resin types, this research evaluates AA2024-T3 aluminium combined with four distinct high-performance fabrics: Innegra (high density polypropylene), Zylon (polybenzoxazole), Basalt, and Para-aramid. The fabric plies were specifically selected for their superior impact performance, a domain currently represented less in FML environmental literature. Furthermore, this work is positioned to capture a detailed manufacturing profile by explicitly incorporating the primary energy demand of metallic surface treatments, lab-scale fabrication cycles, and precision post-processing via water jet cutting. This approach ensures that the influence of material selection and specific manufacturing protocols is systematically captured, providing a foundational baseline for the future scaling of sustainable high-performance composites.

## Methodology

### Materials and fabrication

The materials comprised the fiber fabrics, aluminium alloy sheets and the resin system. The Aramid (K220T2), Zylon (Z300T2) and Innegra (I800P) fabrics were supplied by FIBERMAX Ltd, Greece. The Basalt fabric (Basaltex) and the epoxy resin (Bisphenol A) / hardener (diethylenetriamine) was supplied by CASTRO composites, Spain. The AA2024-T3 sheets (0.5 mm thick) were procured from LAS Aerospace Ltd, United Kingdom. The specifications of the fiber fabrics are given in Table 1. The properties of the different constituent materials are given in Table 2.

**Table 1:** Technical specifications of the fiber fabrics.

Fabric	Weave type	Areal density $\left( \frac{g}{m^2}\right)$	Warp count	Weft count	Fabric thickness (mm)
Aramid (K220T2)	Twill 2/2	220	7.3	7.3	0.31
Basaltex	Twill 2/2	220	7.2	7.2	0.13
Zylon (Z300T2)	Twill 2/2	300	9.2	9.2	0.42
Innegra (I800P)	Plain	800	4	2	1.13

**Table 2:** Mechanical Properties of the fiber fabrics and metallic plies.

Material	Density (g/cc)	Tensile modulus (GPa)	Tensile strength (GPa)
AA2024 T3 skin	2.78	71	446
Epoxy binder	1.16	3.1	45
Aramid (K220T2)		105	3.014
Basaltex	2.67	87	3.1
Zylon (Z300T2)	1.55	270	5.8
Innegra (I800P)	0.84	13.7	0.67

**Fig. 1:**
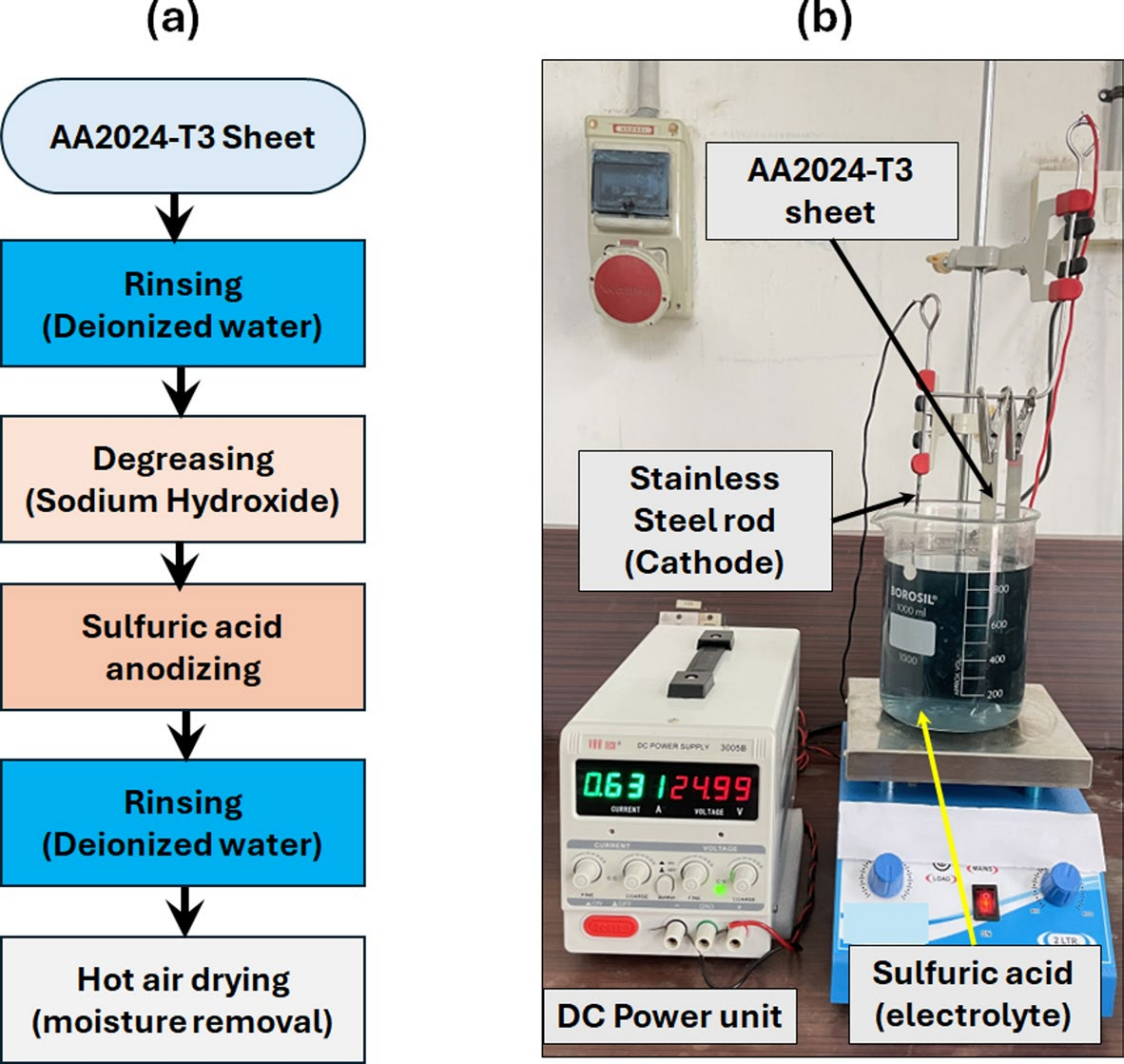
Sulfuric acid anodizing treatment for AA2024-T3 panels (**a**) Schematic representation of stages (**b**) Anodizing setup.

**Fig. 2:**
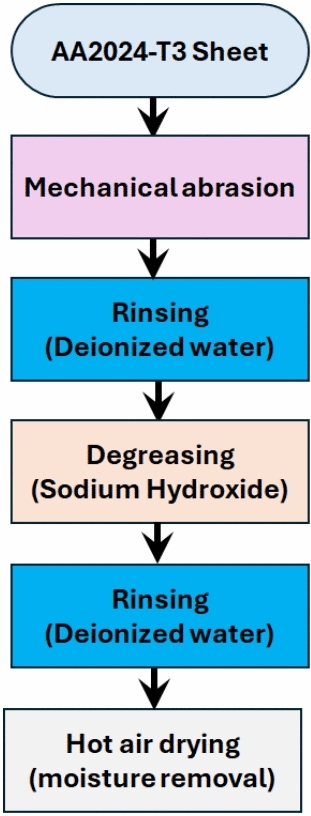
Schematic of the processes in mechanical abrasion for the metallic plies.

Before fabricating the FMLs, the pre-processing stages involved cutting the AA2024-T3 skin to size of 130 mm $\times$ 130 mm and surface preparation to improve the interfacial adhesion between the metallic and fiber-reinforced polymer layers. Surface treatment of the aluminium panels was performed using two methods: sulfuric acid anodizing (SAA) and mechanical abrasion which have been used for surface adhesion improvement of aluminium skins^16,17^. In the SAA process shown in Fig. 1, the AA2024-T3 sheets were pretreated with a 10% sodium hydroxide solution for 10 s, rinsed with deionized water, and dried. Anodizing was carried out using a 0.5 M sulfuric acid electrolyte at a constant voltage of 15 V for 30 min at ambient temperature. The current density was 2 $\frac{A}{dm^2}$. A stainless-steel rod served as the cathode, and the aluminium samples were connected to the anode using stainless-steel clips. The electrolyte was continuously stirred to ensure uniform distribution. Post-anodizing, the skins were rinsed and dried using hot air. In the second surface treatment, mechanical abrasion as shown in Fig. 2 was performed using 60-grit abrasive paper for 5 min along the rolling direction. The abraded surfaces were rinsed, degreased with a 30% sodium hydroxide solution for 10 s, and dried to ensure a clean, moisture-free surface.

**Fig. 3:**
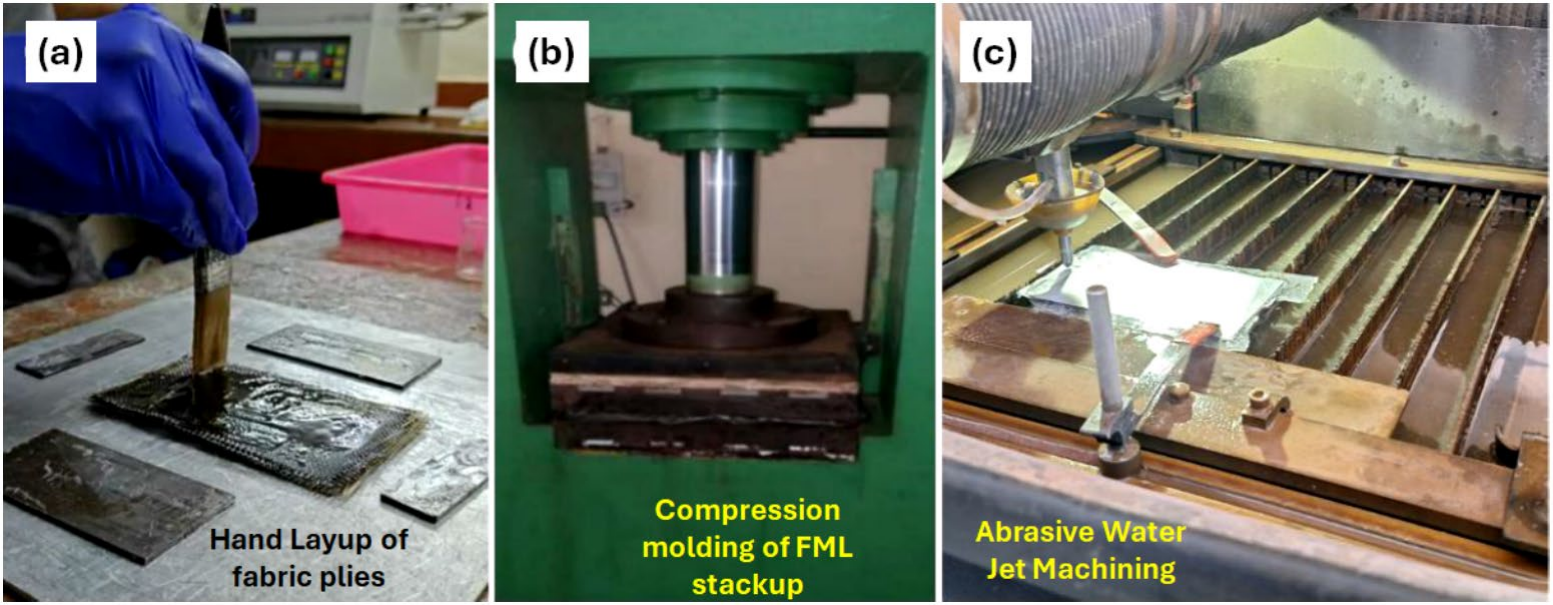
Stages of fabrication of the FML stackups (**a**) Hand Layup of the fabric plies (**b**) Compression molding to desired thickness (**c**) Abrasive water jet machining to create the functional unit.

The stackups of the different FMLs are shown in Table 3. The metallic plies were used as the facing plies sandwiching the core of the fabric layers. The woven fabrics were cut to $130 \times 130$ mm prior to layup. During fabrication of each laminate, flat mould plates were used, with a peel ply placed to prevent sticking of the laminate to the mould plates. Atop the peel ply, the AA2024-T3 plate was placed, and the epoxy resin mixture was applied. The epoxy resin and hardener were mixed in ratio of 75:25. Depending on the configuration, the different fabric plies were successively placed with continuous application of the resin mixture (hand layup technique). Finally, the AA2024-T3 plate was placed on the top of the stack, sandwiching the fabric plies in between two aluminium skins. The mould was then placed within the compression moulding maching followed by cold compression at a pressure of $\sim$ 0.6 MPa. The different stages of the fabrication and post-processing are shown in Fig. 3. The moulding machine, rated at 2.2 kW, was operated for 10 min per stackup, resulting in an energy consumption of approximately 1.32 MJ. Compression was controlled using 1.7 mm precision blocks to ensure uniform laminate thickness.

Post-fabrication trimming to the FU size of $120 \times 120$ mm was performed using AWJM, with an estimated energy consumption of 54 kJ per laminate (based on a 3-minute cutting duration). The parameters used during the machining of the laminates using the AWJM include:

- High performance garnet abrasives (Mesh number $\sim$ 80),
- Mass flow rate of 0.019 $\frac{kg}{m^3}$,
- Water pressure of 30000 psi ($\sim$ 207 MPa)

The fabrication process also involved the use of consumables including cotton waste (32–35 g), acetone for cleaning (5–7 g), rubber gloves (18–20 g), peel ply (2 g), and release agent (2 g), which is taken as unity per FML stackup.

**Table 3:** Details of the different FML configurations.

FML type	Stackup	Metallic ply	Type of fabric and number of layers			
		AA2024-T3	Basalt	Aramid	Innegra	Zylon
AFML-A	Al-Ba$_4$-Al	2	4	–	–	–
AFML-B	Al-Ar$_4$-Al	2	–	4	–	–
AFML-C	Al$_6$	6	–	–	–	–
AFML-D	Al-Zy$_4$-Al	2	–	–	–	4
AFML-E	Al-In$_4$-Al	2	–	–	4	–
AFML-F	Al-Ba$_4$-Al	2$^*$	4	–	–	–

### Life cycle assessment

The present study follows the standard framework of LCA^34,35^, comprising goal and scope definition, inventory analysis, impact assessment, and interpretation of results.

#### Definition

**Fig. 4:**
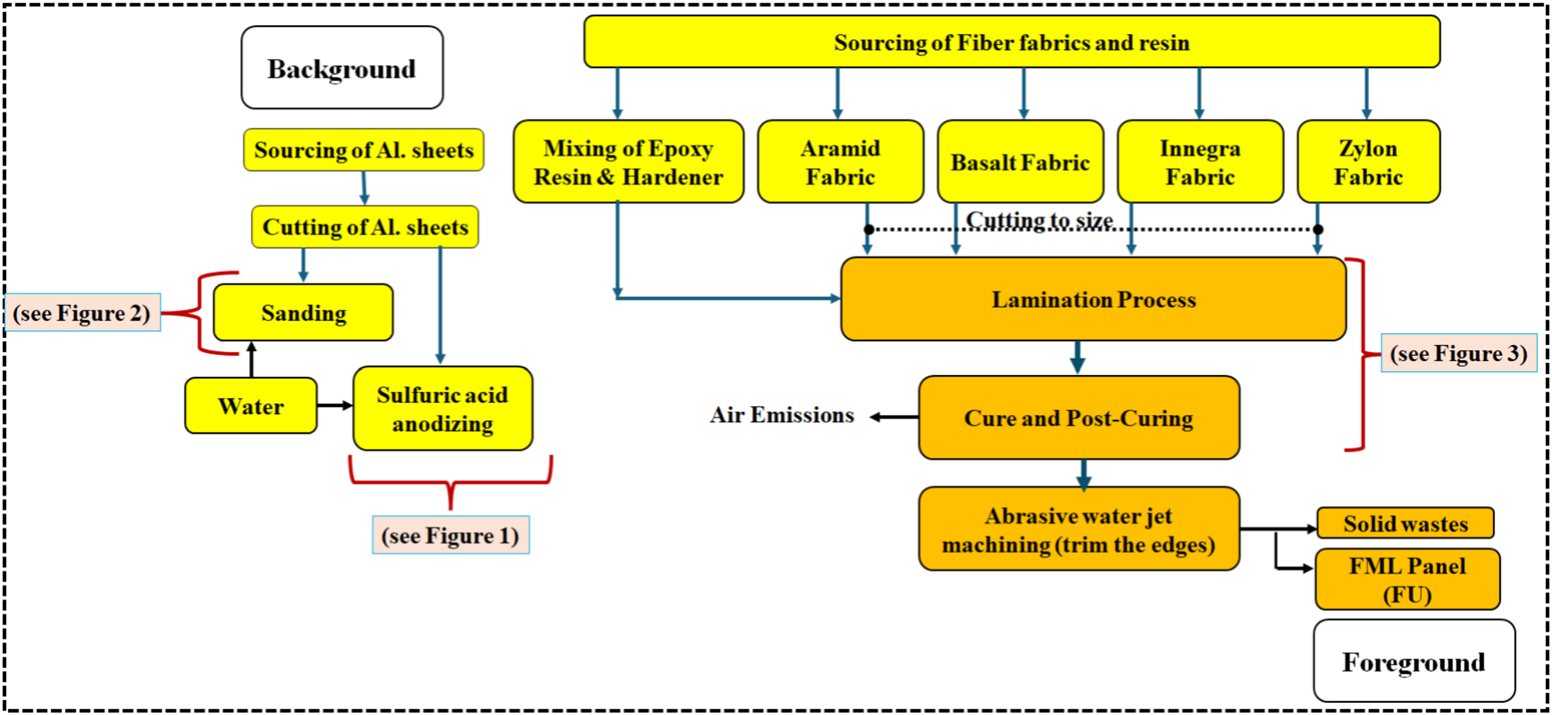
Cradle-to-gate product system for the LCA of different FML stackups, with background processes shown in yellow and foreground processes in orange.

The goal of this LCA is to evaluate the environmental impacts associated with the material requirements and energy consumption during manufacturing of six distinct stackups of FMLs. Each stackup integrates metallic plies (AA2024-T3) with high-performance fabrics-Basalt, Aramid, Innegra and Zylon -in both plain and hybrid configurations, using epoxy resin as the matrix material. Building upon the material processing, fabrication and post-processing, the study adopts a cradle-to-gate approach in compliance with ISO 14040 and ISO 14044 standards employed in similar research^23,32,33^. The system boundaries, encompassing both the Background (material sourcing) and Foreground (lab-scale manufacturing) stages, are schematically represented in Fig. 4. This framework ensures that the primary energy demand and materials usage from the specific processes shown in Figs. 1, 2, and 3 are systematically accounted for in the final environmental profile.

#### Scope

The system boundary for this study was defined as cradle-to-gate, encompassing raw material extraction, constituent manufacturing, and final laminate fabrication. The use-phase and end-of-life (EoL) stages were excluded from the current scope for two primary reasons. First, the primary functional application-ballistic shielding-normally characterized by a stationary or discrete event (impact) may not contribute significantly to direct environmental emissions. Second, excluding speculative use-phase scenarios ensured that the analysis remained focused on the comparative environmental impacts arising strictly from manufacturing variations, such as the different surface treatment protocols (anodizing vs. mechanical abrasion). This boundary provides a transparent, high-resolution view of the manufacturing “environmental investment” required to produce high-performance FMLs before they are integrated into larger structural systems.

#### Functional unit

The functional unit (FU) was defined as an FML panel measuring $120\,\text {mm} \times 120\,\text {mm}$ containing two plies of AA2024-T3 (facing plies or skins) and four fabric plies depending on the type of stackup. The functional unit was defined based on the specimen dimensions required for ballistic impact characterization, justified by the clamping and boundary conditions necessitated for $V_{50}$ ballistic limit determination under the MIL-STD-662F standard^36^. Although the panel specifications were dictated by these standards, the experimental ballistic testing phase is considered outside the scope of the current LCA study. This standardized area allows for a precise comparison of the manufacturing energy and material consumption across the six configurations.

#### Data sources and limitations

 A comprehensive account of raw material inputs, energy consumption, and fabrication processes is provided, along with the assumptions, software tools, and methodological choices employed in the analysis. The inventories of the different product systems were modelled using the OpenLCA 2.5.0 software with the inventory data taken from the databases comprising ecoinvent 3.12 LCIA, Worldsteel2020, USDA$_{1901009}$^37–39^. Since the Ecoinvent database did not contain the data for Zylon, Innegra, basalt, and aramid fibers, new inventories had to be created for the life cycle impact assessment based on similar studies on the synthetic fibers^40^. In light of several identified data gaps, the development of life cycle inventories required some assumptions regarding the time and the energy utilization for the different input materials. Supplier specifications were consulted, and the production stages–including fiber yarn manufacturing, fabric preparation from yarns, and associated processing techniques–were referenced from recent literature and technical documentation.

*Data for the AA2024-T3 skins* The environmental profile for the AA2024-T3 aluminium skins was modeled using a primary production baseline, as the materials were sourced as virgin alloy rather than recycled feedstock. Consequently, the system boundary for the aluminium inventory was defined to encompass the entire upstream sequence, including bauxite extraction, the Bayer process, and Hall-Héroult electrolysis. The AA2024-T3 aluminium alloy sheets are prepared through bauxite mining, alumina refining via the Bayer process, and electrolytic reduction in Hall–Héroult cells, followed by casting, rolling, and heat treatment, with significant energy consumption and waste generation such as red mud and greenhouse gases^41^ (Table 4).

**Table 4:** Life cycle inventory for the production of AA2024-T3 aluminium alloy sheets (120 × 120 × 0.5 mm).

Material	Input material	Mass (g)	Output material	Mass (g)	Process	Energy per ply (MJ)	References
AA2024-T3 (Al–Cu)	Bauxite (Al)	78.50	AA2024-T3 sheet	20.02	Bayer process,	4.20	^42^
	Cu (pure)	0.88	Bauxite residue	40.20	Hall–Héroult,		^43^
	Mg/Mn	0.42	Casting scrap (Aluminium waste)	1.15	Rolling (0.5 mm),		^41^
	Alloying flux	0.25	Emissions	18.68	Heat treatment (T3)		

*Data for the surface treatment of AA2024-T3 skins* The surface preparation of the AA2024-T3 aluminium skins was modeled to include all chemical and energy overheads, as detailed in Table 5. For the sulfuric acid anodizing (SAA) process, the inventory accounts for the consumption of 0.5 M $H_2SO_4$ and 10% *NaOH* pretreatment solutions. The energy demand of 0.45 MJ per panel was calculated based on a constant voltage of 25 V and a current density of 1.5 A/dm$^2$ sustained over 30 min, including mechanical stirring and final hot air drying. The mechanical abrasion pathway, as illustrated in the process flow (Fig. 2), involves the consumption of one 120 mm $\times$ 120 mm sheet of 60-grit emery paper per panel. It is assumed that the total mass of the emery paper is 10.35 g (6.10 g abrasive grit and 4.25 g paper backing). Following abrasion, the panels undergo a sequence of rinsing with deionized water, degreasing with 30% *NaOH*, a secondary deionized water rinse, and final hot air drying for moisture removal. These values serve as approximation estimates for the processing of each $120 \times 120$ mm panel to ensure all ancillary material and energy flows are transparently captured.

**Table 5:** Life cycle inventory data for the surface preparation of AA2024-T3 aluminium skins (per 120 $\times$ 120 mm panel).

Process	Input material	Mass (g)	Output material	Mass (g)	Process	Energy (MJ)	References
Sulfuric acid anodizing (SAA)	H$_2$SO$_4$ (0.5 M)	45.50	Spent electrolyte	45.10	Electrolysis (25V), stirring, rinsing, drying	0.45	^16^
	NaOH (10% soln)	15.00	Aluminium hydroxide	0.15			
	Deionized water	250.0	Wastewater (acidic)	265.25			
Mechanical abrasion	60-grit sand (abrasive)	6.10	Used abrasive grit	6.05	Manual abrasion degreasing, rinsing, drying	0.04	^17^
	Backing paper	4.25	Used backing paper	4.25			
	NaOH (30% soln)	15.00	Degreasing waste	15.05			

*Data for the binder (epoxy)* The epoxy resin system was modeled as a two-part thermosetting matrix consisting of DGEBA resin and TETA hardener. As detailed in Table 6, the 18 g mass per laminate was processed via a cold-curing cycle using a hydraulic compression molding setup. The inventory boundary for the matrix include the primary chemical precursors and the ancillary cleaning solvents, such as acetone, utilized during the handling stage.

**Table 6:** Life cycle inventory data for the epoxy resin system (per FML stackup).

Material	Input material	Mass (g)	Output material	Mass (g)	Ratio	Energy (MJ/kg)	References
Epoxy matrix (18 g per FU)	DGEBA resin	13.50	Cured epoxy matrix	18.00	75	2.88	^44^
	TETA hardener	4.50	Handling waste	0.90	25		^45^
	Acetone	7.00	Volatile emissions	7.00	–		^17^

*Data for the fiber fabrics* Innegra fiber, derived from high-modulus polypropylene, is produced via melt spinning, quenching, and drawing, with inputs including polypropylene resin, electricity, and cooling water, and outputs comprising finished fiber and minor emissions. The fiber is then woven into the fabric of desired specifications^45,54,55^. Energy consumption for Innegra fabric is modeled using a Specific Energy Consumption (SEC) value of $84\text { MJ/kg}$, serving as an approximation estimate for each $120\text { mm} \times 120\text { mm}$ fabric ply. Zylon fiber, chemically known as polybenzoxazole, is synthesized through solution polymerization of aromatic monomers in organic solvents, involving high energy consumption and generating hazardous waste streams, contributing significantly to acidification and human toxicity^56,57^. The energy demand for Zylon is estimated at an SEC of $200\text { MJ/kg}$ per $120\text{ mm} \times 120\text{ mm}$ ply. Basalt fabric is manufactured by melting crushed volcanic rock at temperatures around $1400\text {--}1600^\circ \mathrm{C}$ and extruding it into filaments, with minimal chemical inputs and low emissions, and then weaving into the fabric of desired specifications^58,59^. The energy requirement is calculated using an SEC of $18.5\text { MJ/kg}$ for basalt melting, estimated for each $120\text{ mm} \times 120\text{ mm}$ ply. Aramid fabrics are produced via solution polymerization of aromatic polyamides using petrochemical-derived monomers and organic solvents. Para-aramid fiber inventory was modeled based on the condensation polymerization of 1,4-benzenedicarbonyl dichloride and p-phenylenediamine in an N-methyl-2-pyrrolidone (NMP) solvent system. To ensure chemical accuracy in the LCI, the neutralization of hydrochloric acid byproducts with calcium oxide was accounted for, alongside the energy requirements for the specialized dry-jet wet spinning process necessary for high-tenacity aramid fibers followed by weaving^44^, resulting in high durability but notable environmental impacts from solvent use and volatile organic compound emissions. The energy for aramid is modeled at an SEC of $150\text { MJ/kg}$ as an approximation for each $120\text { mm} \times 120\text { mm}$ ply. The data of the developed life cycle inventories for the four fabrics are tabulated in Table 7.

**Table 7:** Life cycle inventory data for the production of the constituent fiber plies for functional unit size.

Fabric	Input material	Mass (g)	Output material	Mass (g)	Process	Energy per ply (MJ)	Reference
Zylon	p-phenylenediamine	1.60	Zylon (PBO)	4.32	Dry jet wet spinning, weaving	0.86	^46^
	2,6-dihydroxybezoic acid	2.28	Recovered PPA	32.95			^47^
	Polyphosphoric acid	38.77	Waste (non-recoverable)	5.82			
Innegra	Polypropylene	11.75	Innegra (HMPP)	11.52	Melt spinning, drawing, weaving	0.96	^48^
	Hexane solvent	0.35	Recovered hexane	0.33			^49^
	TiCl$_4$ catalyst	0.12	Recovered catalyst	0.11			
			Waste (non-recoverable)	0.26			
Basalt	Basalt rock	3.33	Basalt fabric	3.17	Melt extrusion, sizing, weaving	0.06	^50^
	Sizing agents	0.05	Particulate emissions	0.02			^51^
	Process water	12.50	Waste rock / slag	0.14			
	Nitrogen / air	5.00	Waste water	7.55			
Aramid	1,4-TDP$^*$	2.73	p-aramid	3.17	Condensation polymerization, dry-jet wet spinning, weaving	0.48	^52^
	p-phenylenediamine	1.45	Recovered NMP	11.93			^53^
	NMP solvent	12.70	Calcium chloride	1.48			
	Calcium oxide	0.75	Waste (non-recoverable)	1.05			

*1,4-Terephthaloyl Dichloride

*Data for the fabrication and machining* The life cycle inventory for the fabrication stage was modeled by isolating the cumulative electrical demand from the material constituents. As detailed in Table 8, the processing energy accounts for the 2.2 kW power rating of the hydraulic compression molding machine over a 10-minute duty cycle, totaling 1.32 MJ per FML laminate. Additionally, a 54 kJ allocation was included for the post-fabrication trimming of the stackup to the final $120 \times 120$ mm functional unit size via Abrasive Water Jet Machining. These energy assumptions allow for a granular assessment of the fabrication process’s contribution to the total environmental impact, distinctly separated from the primary production of the fiber reinforcements and metallic phases.

**Table 8:** Process energy and consumables inventory for FML fabrication and post-processing (LCA model assumptions).

Process stage	Parameter/equipment	Input value	Output/waste	Energy (MJ)	References
Cold compression	Hydraulic moulding machine (Duty Cycle: 10 min)	2.2 kW (at 0.6 MPa)	Consolidated FML	1.32	^55^
Post-processing	AWJM trimming (3 min) (to $120 \times 120$ mm)	54 kJ	Kerf slurry waste	0.054	^16^
			Oversize scrap		
Consumables (per FML)	Cotton waste & Gloves	55 g (total)	Solid waste	–	^17^
	Acetone & release agent	9 g (total)	VOC emissions		

*Consolidated airborne emission for the fiber and binder phases* The environmental impact assessment, specifically the carbon footprint analysis, was facilitated by the development of a consolidated airborne emission inventory for the polymer-based constituents. As presented in Table 9, the inventory quantifies the raw emission intensity (kg of gas per kg of material) for the production of Aramid, Zylon, Innegra fibers, and the epoxy resin system. These factors account for primary greenhouse gases (GHGs) such as carbon dioxide ($CO_2$) and methane ($CH_4$), alongside hazardous chemical precursors and byproducts including nitrogen oxides ($NO_x$), phenol, and volatile organic compounds (VOCs). By incorporating these granular emission data, the life cycle model captures the high-energy synthesis requirements and chemical synthesis impacts intrinsic to aerospace-grade high-modulus polymers, ensuring a rigorous cradle-to-gate assessment of the fabricated FML stackups (Table 10).

**Table 9:** Consolidated airborne emission inventory for fiber and resin production (kg/kg of material).

Material	Emission species	Value (kg/kg)	Material	Emission species	Value (kg/kg)
Aramid	Carbon dioxide ($CO_2$)	10.000	Innegra (HMPP)	Carbon dioxide ($CO_2$)	1.800
	Nitrogen oxide ($NO_x$)	0.015		Nitrogen oxide ($NO_x$)	0.001
	Sulphur dioxide ($SO_2$)	0.010		VOCs	0.002
	Methane ($CH_4$)	0.005		Methane ($CH_4$)	0.001
	Sulphuric acid	0.001			
Zylon (PBO)	Carbon dioxide ($CO_2$)	18.000	Epoxy resin	Carbon dioxide ($CO_2$)	3.000
	Nitrogen oxide ($NO_x$)	0.025		Dichloromethane ($CH_2Cl_2$)	0.005
	Phenol	0.005		Nitrogen oxide ($NO_x$)	0.003
	Methane ($CH_4$)	0.008		Phenol	0.002
				Hydrochloric acid (*HCl*)	0.001
				Methane ($CH_4$)	0.001

**Table 10:** Comprehensive airborne emission inventory for the inorganic fiber (basalt) and processes (kg/kg of material)^60^.

Material/Process	Emission species	Value	Material/process	Emission species	Value
Basalt fiber (Melting/Extrusion)	Carbon dioxide ($CO_2$)	2.500 kg/kg	AA2024-T3 (Primary Al alloy)	Carbon dioxide ($CO_2$)	12.500 kg/kg
	Nitrogen oxide ($NO_x$)	0.002 kg/kg		Fluorides ($CF_4, C_2F_6$)	0.001 kg/kg
	Particulates (PM)	0.001 kg/kg		Sulphur dioxide ($SO_2$)	0.012 kg/kg
AWJM cutting (Machining)	Abrasive dust	0.250 kg/FU	Electricity (Grid mix)	$CO_2$-equivalent (Region dependent)	0.6 kg/kWh
	Water vapor	–			

#### Impact categories

The input and output process flows for the fabrication of the different FMLs have been shown in Table 11. Environmental impacts were assessed using midpoint indicators across the following categories: acidification (terrestrial), ecotoxicity (freshwater, marine, and terrestrial), human toxicity (non-carcinogenic), material resources (metals/minerals), and photochemical oxidant formation (human health and terrestrial ecosystems). Inventory data were compiled and processed using standard LCA software and impact assessment methods to quantify the contributions of each material and process to the selected categories. To evaluate the environmental efficiency of the different stacking sequences in a context relevant to high-performance engineering, a normalized impact analysis was performed. In ballistic shielding and aerospace applications, the primary design objective is to achieve maximum energy absorption with a minimum weight penalty, necessitating the use of lightweight armor solutions. Consequently, the absolute environmental impacts were normalized against the areal density (kg/m^2^) of each respective FML configuration. This normalization allows for a functionally equivalent comparison, quantifying the environmental investment required per unit of protective mass, which is critical for the development of sustainable, lightweight shielding structures.

**Table 11:** Process flows including the input and output flows for the FML stackups per FU.

	AFML-A	AFML-B	AFML-C	AFML-D	AFML-E	AFML-F
Inputs (units)						
AA2024-T3 skin	2	2	6	2	2	2
Epoxy (g)	18.1	18.2	22.1	18.5	18.4	18.3
Basalt (g)	12.68	–	–	–	–	12.69
Aramid (g)	–	12.71	–	–	–	–
Zylon (g)	–	–	–	17.28	–	–
Innegra (g)	–	–	–	–	46.09	–
Areal density ($\frac{\mathrm{kg}}{\mathrm{m}^3}$)	3.89	3.85	8.59	4.08	6.16	3.89
Sanding (for Al)	–	–	–	–	–	1
Alkali degreasing (for Al)	1	1	1	1	1	1
Anodizing (for Al)	1	1	1	1	1	–
Consumables during fabrication	1	1	1	1	1	1
Water consumption (kg)	1.19	1.18	1.25	1.21	1.22	0.43
Outputs (units)						
FML laminate (g)	55.95	55.42	123.76	58.72	88.73	55.95
AA2024-T3 waste (g)	5.81	5.65	13.6	5.31	5.39	5.82
Fabric waste (g)	1.39	0.62	–	1.28	1.09	1.38
Acetone waste (g)	5.35	5.25	10.22	12.42	5.25	4.95

#### Component contribution analysis and eco-design leverage

To move beyond a simple comparative ranking and identify specific areas for environmental improvement, a detailed Component Contribution Analysis (CCA) was performed.This analysis establishes the environmental “boundary impacts” by quantifying the percentage contribution of each life cycle stage for the most environmentally burdened configuration (AFML-C: All Anodized Aluminium Plies) and contrasting it with the more favorable (AFML-F: Basalt/Mechanically Abraded Aluminium). These two cases represent the upper and lower impact bounds, respectively, providing a comprehensive envelope for the hybrid configurations investigated.

The system boundary for the CCA remains the defined cradle-to-gate functional unit of the $120 \times 120\text { mm}$ FML panel. The total environmental impact ($I_{\text {total}}$) for a given impact category (e.g., Climate Change) is the sum of the impacts from all primary and secondary components shown in Equation 1 as:

1 $$\begin{aligned} I_{\text {total}} = I_{\text {Al}} + I_{\text {Fiber}} + I_{\text {Resin}} + I_{\text {SurfaceTreat}} + I_{\text {Fabrication}} \end{aligned}$$

The analysis is structured around the following five distinct components:

1. *Aluminium alloy* ($\boldsymbol{I_{\text {Al}}}$): This component includes all impacts associated with the production of the primary AA2024-T3 sheets, from bauxite mining, alumina refining, electrolytic smelting, and rolling.
2. *Fiber *($\boldsymbol{I_{\text {Fiber}}}$): This includes the environmental burdens related to the production of the specific fiber type (e.g., Zylon, Innegra, Basalt), encompassing raw material synthesis and fiber weaving/spinning.
3. *Epoxy resin *($\boldsymbol{I_{\text {Resin}}}$): Impacts from the production of the epoxy polymer and hardener precursors.
4. *Surface treatment *($\boldsymbol{I_{\text {SurfaceTreat}}}$): This is the key process differentiator, quantifying the impacts of either *Anodizing* (chemical inputs, waste treatment, and electricity) or *Mechanical Abrasion* (electricity, water, and sand/abrasive use).
5. *Fabrication processes* ($\boldsymbol{I_{\text {Fabrication}}}$): This covers the energy (thermal and electric) required for the layup, curing cycle (autoclave/VARI), and post-processing steps (e.g., Water Jet Machining for trimming).

The contribution ($C_i$) of each component *i* is calculated as its percentage share of the total impact shown in Equation 2.

2 $$\begin{aligned} C_i = \left( \frac{I_i}{I_{\text {total}}} \right) \times 100\% \end{aligned}$$

## Results and discussion

The Life Cycle Impact Assessment was conducted for the six FML configurations utilizing the ReCiPe 2016 midpoint (H) method to quantify the environmental investment associated with diverse stacking sequences and interfacial treatments. The assessment encompasses a broad spectrum of indicators-specifically terrestrial acidification, climate change, material resource depletion, ecotoxicity, human toxicity, and photochemical oxidant formation-to provide a holistic view of the cradle-to-gate footprint. Table 12 summarizes the environmental impacts of the six stackups across these indicators, representing the absolute cradle-to-gate values prior. These results highlight the inherent environmental trade-offs between configurations dominated by primary metallic layers and those utilizing high-performance synthetic or natural fiber reinforcements.

**Table 12:** Computed environmental impact values for the FML stackups.

Impact category	Unit	AFML-A	AFML-B	AFML-C	AFML-D	AFML-E	AFML-F
Acidification: terrestrial	kg SO2-Eq	0.0312	0.0321	0.0384	0.0324	0.0326	0.0014
Climate change	kg CO2-Eq	1.491	1.492	1.690	1.494	1.495	1.481
Ecotoxicity: freshwater	kg 1,4-DCB-Eq	0.0138	0.0137	0.0173	0.0141	0.0142	0.0004
Ecotoxicity: marine	kg 1,4-DCB-Eq	0.0028	0.0027	0.0029	0.0033	0.0031	0.0003
Ecotoxicity: terrestrial	kg 1,4-DCB-Eq	73.759	73.760	87.695	73.909	74.252	0.004
Human toxicity: carcinogenic	kg 1,4-DCB-Eq	29.970	29.970	33.300	29.970	29.557	29.970
Human toxicity: non-carcinogenic	kg 1,4-DCB-Eq	1.609	1.611	2.353	1.651	1.352	1.610
Material resources: metals/minerals	kg Cu-Eq	20.356	7.734	25.850	7.784	7.711	18.010
Photochemical oxidant formation: human health	kg NOx-Eq	0.109	0.108	0.262	0.107	0.0109	0.106
Photochemical oxidant formation: terrestrial ecosystems	kg NOx-Eq	0.176	0.177	0.422	0.174	0.031	0.175

### Effect of materials in FML configurations

In the six FML configurations, four types of fiber fabrics were utilized, specifically AFML-A (with basalt), AFML-B (with aramid), AFML-D (with zylon), and AFML-E (with Innegra). To distinguish between their responses, the first three impact categories were analyzed.

**Fig. 5:**
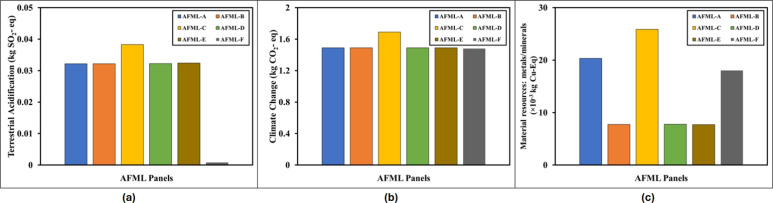
LCA analysis of the AFML stackups (**a**) terrestrial acidification (**b**) climate change (**c**) material resources:metals/ minerals.

Figure 5 illustrates the comparative trends for terrestrial acidification, climate change, and material resource depletion, highlighting the significant influence of constituent mass and processing energy. Regarding terrestrial acidification, the results indicated a negligible variance between FML configurations containing different fiber reinforcements. However, AFML-C, which incorporates six layers of AA2024-T3, exhibited the highest acidification potential. This elevated footprint is primarily attributed to the chemical-intensive anodizing process applied to the expanded metallic surface area, as the production of acidic reagents (sulfuric and chromic acids) is a major driver of acidification. Conversely, the minimum acidification potential was observed in AFML-F. In this configuration, the aluminium skins underwent mechanical abrasion rather than chemical etching, effectively eliminating the reagent-based emission streams that otherwise contribute significantly to the acidification profile. In terms of climate change, the specific fiber type yielded minimal variance in the results, as configurations utilizing different reinforcements exhibited comparable CO$_2$-equivalent values. However, AFML-C displayed a markedly higher global warming potential (GWP), which is fundamentally attributed to the energy-intensive primary production of its six aluminium layers. While the emission factors for high-performance fibers such as Aramid (10.0 kg CO$_2$-Eq/kg) and Zylon (18.0 kg CO$_2$-Eq/kg) are substantially exceeding those of basalt (2.5 kg CO$_2$-Eq/kg) and Innegra (1.8 kg CO$_2$-Eq/kg)-the climate change profile of the FMLs is predominantly governed by the metallic mass fraction. Given the high cumulative mass of the AA2024-T3 skins in the six-layer stack, the embodied energy of the aluminium (12.5 kg CO$_2$-Eq/kg) remains the primary driver of the total GWP, effectively overshadowing the variation in fiber-level impacts across the different configurations. The GWP results obtained in this study are consistent with established benchmarks when adjusted for the functional unit and material mass. Nunez et al.^60^ report a GWP of 16.5 kg CO$_2$-Eq per kg for primary aluminium production globally, and 10.8 kg CO$_2$-Eq for the Rest of the World (RoW) average. When these material-level emission factors are scaled to the specific mass of the AA2024-T3 skins used in the work i.e. $120 \times 120$ mm panels (approximately 0.056 to 0.124 kg depending on the configuration), the resulting impact aligns with the reported values. Furthermore, Braga et al.^32^ reported a GWP ranging from 2.43 to 3.46 kg CO$_2$-Eq for FMLs incorporating aluminium skins and natural fiber reinforcements. While their absolute values are higher than the 1.49 kg CO$_2$-Eq observed for AFML-C, this is justified by their larger functional unit ($300 \times 300 \times 4$ mm), which represents a surface area approximately 6.25 times greater than the specimens in the current study. Consequently, the normalized environmental footprint of the developed FMLs demonstrates a high degree of correlation with existing literature on hybrid metallic-composite systems. Regarding the utilization of material resources, AFML-C exhibited the highest impact, followed by AFML-A and AFML-F, respectively. This trend is primarily driven by the significant consumption of aluminium and basalt reinforcements; as these are inorganic materials derived from geological ores, they contribute substantially to the abiotic depletion of mineral resources. In contrast, configurations AFML-B, AFML-D, and AFML-E showed comparatively lower material resource utilization in terms of mineral depletion. This is attributed to their reliance on synthetic fibers-specifically Innegra (HMPP), Aramid, and Zylon (PBO)-which, as polymer-based materials, shift the environmental burden toward fossil resource depletion rather than the consumption of metallic or mineral ores.

**Fig. 6:**
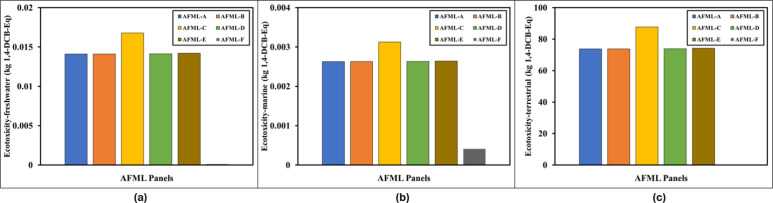
LCA analysis of the AFML stackups (**a**) ecotoxicity:fresh water (**b**) ecotoxicity:marine (**c**) ecotoxicity:terrestrial.

Figure 6 presents the ecotoxicity performance of the six $\text {FML}$ configurations across freshwater, marine, and terrestrial categories, expressed in kg 1,4-DCB-equivalents. The freshwater and marine ecotoxicity profiles exhibited consistent trends across the configurations, with AFML-C demonstrating the highest potential for aquatic toxicity. In contrast, AFML-F showed the lowest values, indicating a significantly reduced environmental burden. While configurations AFML-A, AFML-B, AFML-D, and AFML-E exhibited similar ecotoxicity levels-primarily driven by the primary smelting of the aluminium skins-the marked reduction in AFML-F is attributed to the elimination of chemical surface treatments. In AFML-C, the utilization of six aluminium layers combined with the chemical anodizing process introduces higher concentrations of toxic inorganic salts and acidic effluents into the life cycle inventory. Conversely, the mechanical abrasion employed for AFML-F bypasses these hazardous waste streams, thereby mitigating the discharge of heavy metals and chemical precursors into freshwater and marine ecosystems. Consequently, AFML-F emerges as the most ecologically compatible configuration across all toxicity compartments.

**Fig. 7:**
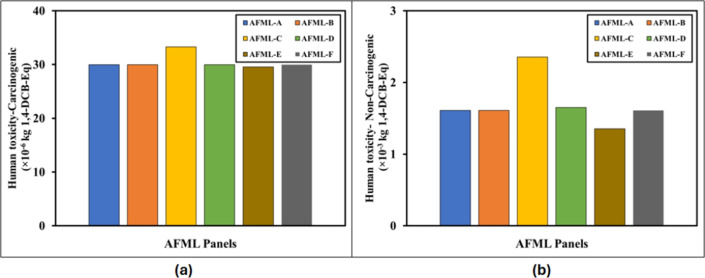
LCA analysis of the AFML stackups (**a**) human toxicity:carcinogenic (**b**) human toxicity:non-carcinogenic.

Figure 7 illustrates the human toxicity impacts of the six FML configurations, categorized into carcinogenic and non-carcinogenic effects. The carcinogenic toxicity values remain relatively consistent across the hybrid configurations, with AFML-C exhibiting slightly elevated levels. This trend is primarily driven by the higher metallic mass fraction in AFML-C, as the primary smelting of AA2024-T3 involves the release of heavy metals and airborne particulates that increase long-term health risks. A similar trend is observed for non-carcinogenic toxicity, where AFML-C again displays the highest potential impact, though the absolute magnitudes are significantly lower than those in the carcinogenic category. Notably, AFML-E (utilizing Innegra or polypropylene fibers) demonstrated the lowest toxicity in both categories. This reduction suggests that the substitution of metallic layers with high-performance synthetic fibers-combined with a reduced reliance on chemical surface treatments-significantly lowers the human health burden by mitigating the release of toxic inorganic substances associated with metal extraction and processing.

**Fig. 8:**
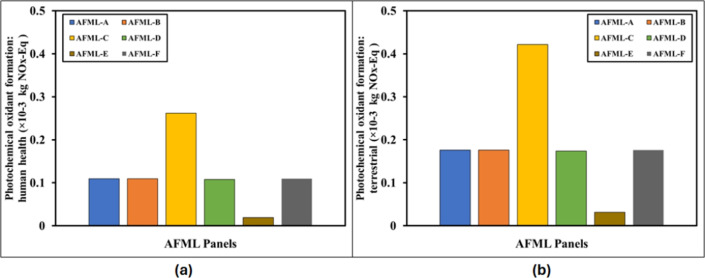
LCA analysis of the AFML stackups (**a**) photochemical oxidant formation:human health (**b**) photochemical oxidant formation:terrestrial.

Figure 8 illustrates the photochemical oxidant formation potential (POFP) for the six FML configurations, evaluated for both human health and terrestrial ecosystem compartments. The highest impacts in both categories are observed for AFML-C, which is attributed to the elevated emissions of ozone precursors, such as nitrogen oxides ($NO_x$) and volatile organic compounds, associated with the intensive primary production and chemical surface treatment of the six-layered aluminium stack. In contrast, lower impact values were recorded for AFML-E (containing Innegra fiber reinforcements). This reduced potential for ground-level ozone formation suggests that the integration of high-performance polyolefin-based fibers-which require less energy-intensive processing compared to both primary aluminium smelting and high-temperature fiber synthesis-mitigates the release of reactive precursors. These trends underscore the critical influence of the metallic mass fraction and the energy intensity of the manufacturing chain on the photochemical oxidant profile.

### Normalized impact of the FMLs

**Fig. 9:**
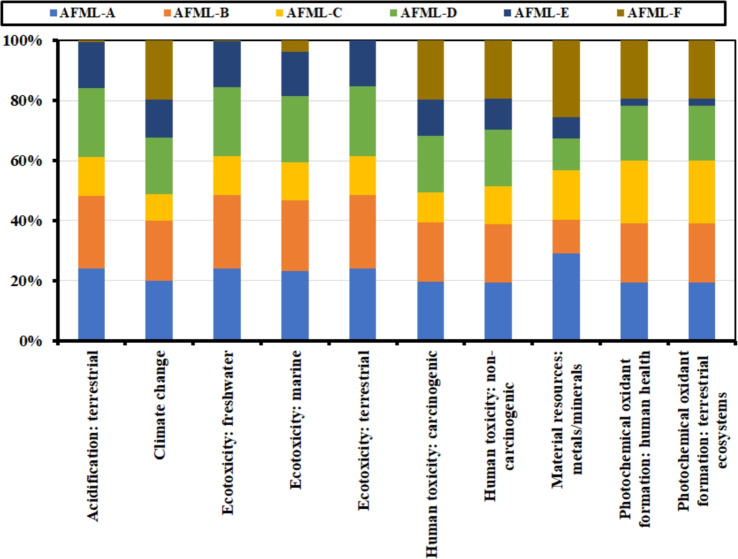
Normalized life cycle impact assessment of the six $\text {FML}$ configurations across ReCiPe 2016 impact categories using areal density as the normalizing parameter.

To provide a rigorous comparative analysis that accounts for the varying weight of the stacking sequences, the environmental impacts were normalized by areal density (kg/m^2^) of each FML. As presented in Table 13, this performance-based metric reveals a shift in the environmental hierarchy. While AFML-C exhibited the highest absolute burden, its normalized climate change and toxicity values are lower than the fiber-dominant hybrids, reflecting a higher environmental efficiency per unit mass for the metallic-heavy configuration. In contrast, the zero-value acidification and terrestrial ecotoxicity for AFML-F persist after normalization, reinforcing the environmental superiority of mechanical surface treatments regardless of material weight (Fig. 9).

**Table 13:** Tabulated data of the Normalized environmental impact of the FML stackups using areal density as the parameter (per $\frac{kg}{m^2}$).

Impact category	AFML-A	AFML-B	AFML-C	AFML-D	AFML-E	AFML-F
Normalized acidification: terrestrial	0.008	0.008	0.004	0.008	0.005	0.000
Normalized climate change	0.383	0.387	0.173	0.365	0.242	0.381
Normalized ecotoxicity: freshwater	0.004	0.004	0.002	0.003	0.002	0.000
Normalized ecotoxicity: marine	0.001	0.001	0.000	0.001	0.000	0.000
Normalized ecotoxicity: terrestrial	18.984	19.165	10.204	18.125	12.050	0.001
Normalized human toxicity: carcinogenic	7.713	7.787	3.875	7.350	4.797	7.709
Normalized human toxicity: non-carcinogenic	0.415	0.418	0.274	0.405	0.219	0.414
Normalized material resources: metals/minerals	5.239	2.010	3.008	1.909	1.251	4.635
Normalized photochemical oxidant formation: human health	0.028	0.029	0.031	0.026	0.003	0.028
Normalized photochemical oxidant formation: terrestrial ecosystems	0.045	0.046	0.049	0.043	0.005	0.045

The normalized impacts categorized by fabric type, as presented in Table 13, reveal a distinct trade-off between mineral extraction and processing efficiency. AFML-A and AFML-F, utilizing four layers of basalt fiber, exhibit the highest normalized material resource depletion (up to 5.239 per kg/m^2^), reflecting the substantial mineral footprint associated with geological ore extraction. In contrast, the synthetic configurations-specifically AFML-E (Innegra) and AFML-D (Zylon)-demonstrate superior mineral efficiency, with AFML-E achieving the lowest value of 1.251 per kg/m^2^. Furthermore, the corrected photochemical oxidant formation values for AFML-E (0.003 for human health) indicate that the integration of Innegra fibers significantly mitigates atmospheric precursor emissions compared to other configurations. While AFML-C appears more efficient in climate change and carcinogenic toxicity due to its high mass-to-area ratio, the zero-value acidification and negligible terrestrial ecotoxicity for AFML-F persist after normalization, reinforcing the environmental superiority of mechanical surface treatments across different fabric types.

*Highlights of the component contribution analysis* The Component Contribution Analysis provides critical insight by translating macro-level comparative LCA results into actionable eco-design strategies (refer Table 14). The analysis reveals that Global Warming Potential is predominantly governed by the aluminium supply chain and electricity consumption, which together account for over 70% of the total impact in both AFML-C and AFML-F. Specifically, the aluminium contribution remains high in both cases (40.53% and 45.58%, respectively), underscoring that the primary metallic mass remains the fundamental carbon driver regardless of the stacking sequence. However, a strategic shift in environmental hotspots is observed in the acidification and human toxicity categories. For AFML-C, surface treatment is the primary hotspot-contributing 73.19% to the acidification potential-due to the chemical reagents and acidic effluents inherent to the anodizing process. In contrast, this impact is entirely mitigated in AFML-F through the adoption of mechanical abrasion. Consequently, the acidification profile of AFML-F shifts toward the upstream emissions of aluminium smelting, specifically the sulfur dioxide ($SO_2$) and nitrogen oxide ($NO_x$) precursors generated during ore reduction. Furthermore, the human toxicity of AFML-F is largely dictated by the electricity grid (70.29%), suggesting that once chemical processing is eliminated, further environmental optimization must focus on transitioning to renewable energy sources and utilizing secondary (recycled) aluminium.

**Table 14:** Component contribution analysis (CCA) results: percentage share of total environmental impact for critical categories.

FML configuration	Impact category	Aluminium [%]	Fiber [%]	Resin [%]	Surface treatment [%]	Fabrication [%]	Electricity [%]
AFML-C	Acidification potential	13.170	0.235	0.287	73.190	0.052	13.065
	Climate change	40.531	6.686	3.905	0.592	3.550	32.958
	Human toxicity	70.549	0.000	1.351	19.514	0.090	8.496
AFML-F	Acidification potential	49.119	8.806	10.763	0.000	1.957	29.354
	Climate change	45.577	7.630	4.456	0.675	4.051	37.610
	Human toxicity	26.083	1.832	1.499	0.167	0.133	70.286

## Conclusions

This study successfully implemented a comprehensive cradle-to-gate Life Cycle Assessment utilizing OpenLCA 2.5.0, integrated with the ecoinvent 3.12, Worldsteel 2020, and USDA databases. By applying the ReCiPe 2016 (H) midpoint method, the environmental investment of six distinct FML configurations was systematically quantified. The assessment demonstrates that fiber selection and surface preparation methods are significant determinants of the total environmental impact, supplementing the influence of the material type. These results provide a robust framework for sustainable composite design, proving that the strategic selection of mechanical surface preparation and synthetic fiber substitution can significantly decouple structural performance from environmental burden.

- Among the FMLs, AFML-F exhibited the most favorable environmental profile across the assessed categories, which was attributed to the replacement of chemical anodizing with mechanical abrasion and the use of basalt fiber reinforcements (natural and inorganic), resulting in near-zero acidification potential and minimal terrestrial ecotoxicity.
- AFML-C (with six metallic AA2024-T3 plies) displayed the highest absolute impacts in climate change, human toxicity, and material resource depletion, primarily driven by the high cumulative energy demand primary AA2024-T3 aluminium and the hazardous effluents associated with surface treatments.
- The chemical surface treatment aluminium plies was identified as a dominant environmental bottleneck, with the component contribution analysis revealing that anodizing accounts for 73.19% the total acidification potential in chemically treated stacks.
- The component contribution analysis demonstrated a strategic shift in environmental hotspots; while impacts in AFML-C are largely process-driven at the fabrication stage, the burden in the optimized AFML-F configuration shifts toward the upstream electricity grid, which accounted for 70.29% the human toxicity indicator.
- Selecting low-impact fiber reinforcements and substituting energy-intensive chemical treatments with mechanical alternatives is essential for minimizing the environmental footprint FMLs while maintaining structural performance for shielding applications.

### Future scope

The future FML development, guided by LCA findings, must prioritize integrated eco-design strategies across the entire product lifecycle. A key area for investigation is the substitution high-impact constituents, moving beyond energy-intensive synthetic fibers toward bio-based reinforcements or secondary (recycled) fibers to further mitigate climate change impacts. Critically, future research should expand upon the optimization interfacial bonding by replacing conventional chemical treatments with scalable, low-impact sustainable alternatives that minimize acidification and human toxicity burdens. Furthermore, a significant research gap remains in the scaling laboratory-scale data to industrial-scale manufacturing. Future prospective LCAs should investigate how transitions from manual batch processing to automated, continuous industrial lines affect environmental efficiency, particularly through improved material yield and reduced energy intensity per unit area. This includes the evaluation energy-saving, out–autoclave (OOA) processes and design-for-disassembly frameworks that facilitate the end–life separation and recovery metal and composite plies. Bridging the gap between laboratory results and industrial-scale implementation will be essential for moving FML technologies toward a commercially viable circular economy model.

## Data Availability

Data supporting the findings of this study are available from the corresponding author upon reasonable request.
